# Notes and Formulæ

**Published:** 1889-05

**Authors:** 


### PRACTICAL NOTES AND FORMULÆ.

---

**Perfect Antiseptic.**—(Rotter.)

| | | |
|---|---|---|
| ℞. | Corrosive sublimate . . . . | 5 parts |
| | Chloride of sodium . . . . | 25 " |
| | Phenic acid . . . . . . | 200 " |
| | Chloride of zinc . . . . | 500 " |
| | Sulpho phenate of zinc . . . | 500 " |
| | Boric acid . . . . . | 300 " |
| | Salicylic acid . . . . . | 60 " |
| | Thymol . . . . . . | 10 " |
| | Citric acid . . . . . . | 10 " |
| | Water . . . . . | 100,000 " |

This makes the author's strong solution. To obtain a weak solution, leave out the sublimate and phenic acid. The solution remains clear and transparent. Steel instruments are not acted upon by it.—*Ib.*

**Irritable cough in Phthisis with night-sweats.**—

| | | |
|---|---|---|
| ℞. | Tinct. opii deodorat. . . . | ʒ ij |
| | Acidi sulph. dil. . . . | ʒ j m xij |
| | Syrupi pruni. Virg. . . | ad ℥ iij |

M. et Signa.—A teaspoonful when the cough is troublesome.

*Woodbury.*

**Mistura Antifebrilis.**—Each tablespoonful contains:

| | |
|---|---|
| Morphiæ acetatis, . . . . . | gr. ⅛ |
| Acidi acetici dil., . . . . . | 5 min. |
| Tr. aconiti, . . . . . . | 1½ to 3 drops |
| Spts. etheris nitrosi, . . . . | |
| Syrupi limonis, . . . . . . | ää 1 dr. |
| Liq. ammon. acetat, . . | q. s. ad. 4 ℥. |

M. Dose, a tablespoonful.—*Ib.*

**Anæsthetic Mixtures.**—Mr. Geo. M. Foy has been writing a series of articles on anæsthetics for the *Dublin Journal of Medical Science* from which we extract the following:

1. A. C. E. Mixture.

| | |
|---|---|
| Alcohol sp. gr. .838. . . . . . | 1 part. |
| Chloroform sp. gr. 1.497. . . . | 2 parts. |
| Ether sp. gr. .753. . . . . . | 3 parts. |

2. Martindale and Westcott's Mixture.

| | |
|---|---|
| Absolute Alcohol sp. gr. 0.795. . . | 1 volume. |
| Chloroform sp. gr. 1.498. . . . . | 2 volumes. |
| Ether sp. gr. 0.720. . . . . | 3 volumes. |

3. Billroth's Mixture.

| | |
|---|---|
| Chloroform . . . . . . . | 3 parts. |
| Alcohol, Ether } of each . . . . . | 1 part. |

4. The Vienna Mixture.

| | |
|---|---|
| Chloroform . . . . . . | 1 part. |
| Ether . . . . . . . | 3 parts. |

By weight, Mix.

5. Buxton's Formula for Methylene.

| | |
|---|---|
| Alcohol . . . . . . . | 1 part. |
| Chloroform . . . . . . . | 4 parts. |

Mix.

To diminish the risk in cases of cardiac asthenia, the addition of nitrite of amyl to chloroform in the proportions of two drachms of the former to a pound of chloroform was recommended; the combination, however, did not become popular and is never used.—*Ib.*

**Vomiting of Pregnancy.**—In cases of incorrigible vomiting due to pregnancy, M. Hubert (*Lyon Medical*) recommends the following:

| | | |
|---|---|---|
| ℞. | Tinctur. Iodini . . . . | gtt. vj. |
| | Kali Iodidi . . . . . | ʒ jss. |
| | Aquæ Destillat . . . . | ℥ ivss. |

M. ft. sol. Sig: A tablespoonful three times a day.

**Asthma.**—(*Germain See*):

℞. Pyridin, . . . . . 1 dr.

Sig.—Put on a hot plate in a small room, and send patient to inhale vapor several times.—*Ib.*

